# Exploring the Relationship Between Empathy, Self-Construal Style, and Self-Reported Social Distancing Tendencies During the COVID-19 Pandemic

**DOI:** 10.3389/fpsyg.2021.588934

**Published:** 2021-02-15

**Authors:** Carl Michael Galang, Devin Johnson, Sukhvinder S. Obhi

**Affiliations:** Social Brain, Body and Action Lab, Department of Psychology, Neuroscience and Behaviour, McMaster University, Hamilton, ON, Canada

**Keywords:** COVID-19, empathy, self-construal style, social distancing, mask use, correlations

## Abstract

Social distancing has become the most prominent measure many countries have implemented to combat the spread of COVID-19. The aim of the current study was to explore the potential role of empathy and self-construal styles, as individual personality traits, on self-reported social distancing. Participants completed the Interpersonal Reactivity Index (a multi-dimensional measure of trait-levels of empathy), the Singelis Self-Construal Scale (a measure of self-construal styles), and were asked to rate their level of social distancing and how much they endorsed social distancing on a five-point Likert-scale. Across a large and diverse sample (with participants collected from Canada, United Kingdom, Sweden, and United States; total *n* = 967), results showed that trait-levels of empathic concern (EC) and perspective taking (PT) positively correlates with social distancing. However, we did not find evidence to suggest that trait-levels of personal distress correlates with social distancing. We interpret these findings as suggesting that empathy, both its altruistic (EC) and cognitive (PT) dimensions, plays an important role in motivating people to socially distance and should be emphasized during times of crisis. Furthermore, we suggest that emphasizing a person’s self-distress during times of crisis may not be an effective approach in promotion social distancing policies (or other prosocial behaviors). We also found that *both* independence and interdependence self-construal styles positively correlates with social distancing. While we expected the latter result, we did not expect the former. This suggests that more work is needed to fully understand how self-construal styles, along with their cultural level analogs (i.e., Individualism-Collectivism), influences social distancing. Overall, these results provide us with novel multi-national data about the role of individual differences on social distancing tendencies specifically, and human behavior during a global health crisis more generally.

## Introduction

Citing the alarming levels of spread and severity, on March 11th, 2020, the World Health Organization (WHO) officially declared COVID-19 as a pandemic^1^. Even before this official declaration, governments and organizations around the world were already proposing various policy implementations to combat the spread of COVID-19, the most prolific being the promotion and regulation of social distancing. While exact definitions of social distancing differ by region, the general idea is that physical contact with other people should be kept to a minimum. At a societal level, this has resulted in businesses such as bars and gyms closing, for markets to implement customer movement and distancing policies, for schools to switch to an online format, and in general, for people to stay home as much as possible. Although social distancing is actively promoted by the WHO and various government bodies around the world, there appear to be individual differences in the extent to which people engage in social distancing behavior. In extreme cases, there have been large-scale protests against government-imposed lockdowns of businesses and schools in the United States^2^. As such, an interesting and pertinent question is: what factors lead individuals to engage in social distancing behaviors? The current study addresses this issue by exploring the potential role of two factors on social distancing: empathy and self-construal style.

### Empathy

Empathy is colloquially defined as the ability to share and understand the emotional states of others (e.g., Preston and de Waal, 2002; Bird and Viding, 2014; Coll et al., 2017), and is often considered to be a primary motivator for prosocial behaviors (e.g., Batson, 2011; Davis, 2015; Decety et al., 2016); for example, trait-levels of empathy (that is, empathy as a particular disposition) have been linked to prosocial behaviors in resource allocation tasks (e.g., Galang and Obhi, 2020; Thielmann et al., 2020). Given that social distancing can be thought of as a prosocial behavior, in the sense that one sacrifices certain comforts and obligations (e.g., going to a movie with friends) for the overall good of the group, then it stands to reason that trait-levels of empathy should also positively correlate with social distancing (although it is also conceivable that *not* socially distancing in order to comfort others who are alone/scared may be considered prosocial–discussed more below in sections “Materials and Methods” and “Discussion”); and indeed, very recent work by Pfattheicher et al. (2020) has shown that, across three different samples obtained from United States, United Kingdom, and Germany, self-reported levels of empathy were a significant and positive predictor of self-reported social distancing behavior. Furthermore, they found that inducing empathy promotes motivation to both socially distance and wear masks in public spaces.

However, it is important to note that Pfattheicher et al. (2020) measurement of self-reported empathy used three items: “I am very concerned about those most vulnerable to coronavirus (COVID-19),” “I feel compassion for those most vulnerable to coronavirus (COVID-19),” and “I am quite moved by what can happen to those most vulnerable to coronavirus (COVID-19).” This particular measure seems to operationalize empathy as a single construct and is specifically embedded in the context of COVID-19. As such, it is still an open question whether empathy, measured as a multi-dimensional stable personality trait (rather than as something specifically related to COVID-19), is associated with social distancing. Furthermore, without a measure of self-distress, it remains to be seen whether self-oriented motivations (e.g., social distancing to protect one’s own health, rather than as a prosocial act *per se*) are also influencing social distancing behaviors. To answer these questions, we opted to utilize the most commonly used measure of trait-levels of empathy: The Interpersonal Reactivity Index (IRI); (Davis, 1980, 1983).

The IRI consists of four subscales: Perspective Taking (PT), Empathic Concern (EC), Personal Distress (PD), and the Fantasy Scale (FS)–PT reflects the tendency or ability to adopt the point of view of other people, EC reflects the tendency to experience feelings of warmth, compassion and concern for others undergoing negative experiences, PD reflects the amount of discomfort and anxiety that occurs as a result of observing the negative experiences of others, and lastly, FS reflects the tendency to transpose or identify strongly with fictional characters (in movies, plays, books, etc.). Based on these definitions, PT and EC seem to tap into different aspects of empathy, with PT seemingly targeting the more cognitive aspects of empathy while EC seemingly targeting a person’s altruistic disposition (and the accompanying emotional experiences). Contrary to PT and EC, Davis (1983) is clear that PD is *not* a measure of empathy, as a focus on one’s own discomfort and anxiety when observing another’s negative experience is likely to interrupt empathic processes (also see: Bird and Viding, 2014). However, PD lends itself well as a measure of self-distress, which we have used in this study to test whether self-oriented motivations, instead of (or perhaps in conjunction with) empathy, predicts social distancing. Lastly, as FS is not an empathy measure *per se*, we do not include it in our analysis (although participants completed the scale as it is a part of the 28-item IRI questionnaire).

### Self-Construal Style

In addition to empathy, another individual trait that may predict social distancing is self-construal style. Self-construal style refers to how “individuals define and make meaning of the self” (pg. 143; Cross et al., 2011). Based on Markus and Kitayama (1991) seminal work, self-construal style is often thought to be made up of two components (although others exist; see Cross et al., 2011): Independent Self-Construal and Interdependent Self-Construal. In short, people high on independent self-construal will emphasize their uniqueness and separateness from others, while people high on interdependent self-construal relate the self to their role in particular in-groups. Van Bavel et al. (2020) have recently suggested that an emphasis on interdependent self-construal may be beneficial in coordinating efforts to socially distance as individuals may prioritize obligations and duty over personal desires. Providing evidence for this claim, Biddlestone et al. (2020) have recently reported that self-reported levels of collectivism belief positively correlated with social distancing intentions. Although the distinction between collectivism and interdependence self-construal is debated (e.g., Cross et al., 2011), it is common to treat the two constructs as the same but at different levels of analysis: collectivism at the cultural level and interdependence at the individual level (with the same relationship between individualism and independence). And as far as we are aware, no study to date has explicitly explored the relationship between self-construal style and social distancing behavior. Finding that interdependence is a positive and significant predictor of social distancing would not only corroborate Biddlestone et al. (2020) findings, but also provide further evidence that different policy strategies may be needed for different cultural/regional contexts.

To measure self-construal style, we opted to utilize the Singelis Self-Construal Scale (SCS) (Singelis, 1994). The SCS has two subcomponents: Independence (Ind) and Interdependence (Int), measuring independence and interdependence self-construal, respectively. Singelis (1994) emphasizes that Ind and Int should be treated as separate components, rather than as a single component at two ends of a spectrum. As such, it is possible to find that *both* self-construal styles positively predict social distancing behavior. Indeed, finding that Ind also correlates with social distancing may suggest that multiple strategies can be implemented in promoting social distancing behaviors (e.g., emphasizing how one’s personal goals may be disrupted by *not* social distancing).

### The Current Study

The aim of the current study is to test whether trait levels of empathy and self-construal style are correlated with self-reported levels of social distancing. Note that our measure of social distancing is taken from Pfattheicher et al. (2020), wherein participants are asked to answer the question: “Because of coronavirus COVID-19, I am massively curtailing social contact (so-called ‘social distancing’).” on a 1–5 Likert-scale. Given it is possible that some participants may not be in control of their social distancing *behaviors* (e.g., they are forced to go into work due to the nature of their job; they have interpersonal commitments to see family and friends who are scared/alone), we opted to also measure social distancing *belief* via the question: “I believe that social distancing is the right course of action.”

We can make a few predictions based on previous studies. First, given Pfattheicher et al. (2020) results, we can predict that EC will positively correlate with self-reported levels of social distancing behavior and belief (as Pfattheicher et al. (2020) empathy questions seems to tap into the same construct as EC). However, as this study is the first to test the relationship between PT/PD and social distancing, we do not make any strong predictions regarding these factors. Second, given Biddlestone et al. (2020) results, we can predict that Int will positively correlate with social distancing behavior and belief. Biddlestone et al. (2020) also report that individualism negatively predicted intentions to engage in social distancing; this suggests that we might also find the same negative correlation between Ind and social distancing behavior and belief.

## Materials and Methods

### Participants

All participants were collected via SONA or Prolific, both of which are online platforms used to recruit participants for studies (with SONA consisting of students enrolled in various psychology courses at McMaster University, whereas Prolific allows the recruitment of a general sample from the community). We initially aimed to collect *n* = 193 based on an *a priori* power analysis via G^∗^Power (Faul et al., 2007, 2009), which showed *n* = 193 is needed to find *r* = 0.2 at 80% power. However, in our Canadian student sample (collected via SONA for course credit), we were only able to collect *n* = 176 (mean age 19.4; female = 118, male = 57, non-binary = 1) before the summer student pool was exhausted. Note that we did not implement any exclusion criteria for this sample. For our community samples, we used Prolific to recruit participants (all participants were paid £1.60, at a rate of £6.40/h) as there is evidence to suggest that samples obtained from this platform are superior compared to MTurk and other alternative platforms (e.g., Peer et al., 2017; Palan and Schitter, 2018). In these cases, we rounded up our sample size to 200 and filtered participants both by Nationality and “Current Country of Residence.” This sample size was met with our United Kingdom (mean age = 35.9; female = 156, male = 41, N/A = 3), New York (mean age = 34.5; female = 87, male = 108, non-binary = 3, N/A = 2), and Florida (mean age = 38.7; female = 83, male = 113, non-binary = 3, N/A = 1) samples; however, we only obtained *n* = 191 for our Swedish (mean age = 28.4; female = 45, male = 145, N/A = 1) sample before signups from the pool were exhausted. Note that, other than Nationality and Current Country of Residence, no other exclusion criteria were used for our Prolific samples. Overall, then, our total sample size reached 967 before the end of data collection. A sample size of 967 is sensitive enough to detect *r* = ∼0.09 at 80% power (although note that sample size fluctuates depending on the number of blank answers on the scales–see section “Data Analysis Plan” below).

The participant ethnicity breakdown per region is as follows: Canada–63 White, 41 South Asian, 38 East Asian, 13 Middle Eastern, 6 Black, 1 Hispanic/Latino, 1 Pacific Islander, 0 Native American, 10 “Other,” 3 N/A; United Kingdom–173 White, 6 South Asian, 5 East Asian, 2 Middle Eastern, 6 Black, 0 Hispanic/Latino, 0 Pacific Islander, 0 Native American, 4 “Other,” 4 N/A; Sweden–169 White, 4 South Asian, 2 East Asian, 4 Middle Eastern, 4 Black, 1 Hispanic/Latino, 0 Pacific Islander, 1 Native American, 5 “Other,” 1 N/A; New York–132 White, 4 South Asian, 15 East Asian, 0 Middle Eastern, 26 Black, 11 Hispanic/Latino, 0 Pacific Islander, 2 Native American, 7 “Other,” 3 N/A; Florida–152 White, 3 South Asian, 5 East Asian, 0 Middle Eastern, 16 Black, 17 Hispanic/Latino, 0 Pacific Islander, 0 Native American, 4 “Other,” 3 N/A.

The Canadian sample recruitment started on May 19th, 2020 and ended on July 11th, 2020. The United Kingdom sample was fully collected on June 24th, 2020. The Swedish sample recruitment started on June 24th, 2020 and ended on July 13th, 2020. Both the New York and Florida sample were collected over July 3rd and 4th, 2020. Regarding the rate of new cases during the collection periods: New York was in a period of declining new cases (Dong et al., 2020) having come off a period in which New York City was declared the global epicenter of the virus (Thompson et al., 2020). Florida made steady increases in new cases (Dong et al., 2020) reaching record highs during the point of collection (Andone and Maxouris, 2020). Per data from the WHO, start and end dates for data collection in Sweden were indicated by daily increases when focusing on a rolling 7-day average (WHO, 2020). Canada showed a daily decrease in cases at the start of data collection but a daily increase by the end of data collection on July 11th (WHO, 2020). Finally, at the point of data collection, United Kingdom officials reported a daily decrease in cases (WHO, 2020).

### Materials and Procedure

Participants completed all questionnaires via LimeSurvey. Participants first read a letter of information and gave their consent to take part in the study. They then answered questions regarding age, gender, and ethnicity. Trait levels of empathy were assessed using the IRI (see Davis, 1980 for the development and validation of this questionnaire). The IRI consists of four subscales: PT, EC, PD, and FS (described in section “Introduction”). To measure SCS, we used the Singelis SCS (see Singelis, 1994 for the development and validation of this questionnaire). The SCS has two subcomponents: Independence (Ind) and Interdependence (Int), measuring Independence and Interdependence Self-Construal Styles, respectively (described in the introduction).

Lastly, our measure of social distancing behavior is taken from Pfattheicher et al. (2020), wherein participants are asked to answer the question: “Because of coronavirus COVID-19, I am massively curtailing social contact (so-called ‘social distancing’).” on a 1–5 Likert-scale. Given it is possible that some participants may not be in control of their social distancing behaviors (e.g., they are forced to go into work due to the nature of their job; they have interpersonal commitments to see family and friends who are scared/alone), we also opted to also measure social distancing *belief* via the question: “I believe that social distancing is the right course of action.” Finally, included in our New York and Florida samples, participants were asked equivalent questions regarding mask use: “Because of coronavirus COVID-19, I am wearing a face mask outside of my home.” and “I believe that wearing a face mask outside of my home is the right course of action.”

In the Canadian, United Kingdom, and Swedish samples, participants always completed the questionnaires in the following order: IRI → SCS → Social Distancing Questions. In the New York and Florida samples, the order was fully randomized and included the Mask Questions. At the end of the study, participants were provided with a debrief form and were sent back to SONA or Prolific to confirm the completion of the study.

### Data Analysis Plan

Due to the negatively skewed distribution of the social distancing data (suggesting that most participants answered a 4 or 5 on the Likert-scale), we opted to use Spearman’s rho (a non-parametric alternative to Pearson’s r) between each of the IRI (excluding FS) and SCS subscales and social distancing scores (behaviors and beliefs). This leads to five trait measures (PT, EC, PD, Ind, and Int) and two social distancing measures (social distancing behavior and belief); thus, our overall analysis contained ten possible correlations between the trait measures and social distancing scores. To control for the inflation of our type 1 error rate, we set our false discovery rate (FDR) to 0.05 and report corrected *p*-values (Benjamini-Hochberg Adjusted). Please note that participants were free to *not* answer any question they did not want to. As such, some subscales were left blank and sample size fluctuates as a result. We report sample size per measure for transparency. We also report both original and adjusted *p*-values. A similar analysis is conducted for mask use (as the data showed the similar distribution to the social distancing scores).

## Results

### Social Distancing

Our analysis showed that EC significantly and positively correlated with Social Distancing Behavior [rho = 0.17, *p* < 0.001, *p*_*adjusted*_ < 0.001, *n* = 947] and Belief [rho = 0.22, *p* < 0.001, *p*_*adjusted*_ < 0.001, *n* = 947]. PT also significantly and positively correlated with Social Distancing Behavior [rho = 0.07, *p* = 0.032, *p*_*adjusted*_ = 0.04, *n* = 945] and Belief [rho = 0.08, *p* = 0.016, *p*_*adjusted*_ = 0.022, *n* = 943]. Lastly, both Ind and Int significantly and positively correlated with Social Distancing Behavior [Ind: rho = 0.13, *p* < 0.001, *p*_*adjusted*_ < 0.001, *n* = 927; Int: rho = 0.12, *p* < 0.001, *p*_*adjusted*_ < 0.001, *n* = 911] and Belief [Ind: rho = 0.08, *p* = 0.012, *p*_*adjusted*_ = 0.02 *n* = 928; Int: rho = 0.09, *p* = 0.006, *p*_*adjusted*_ = 0.012, *n* = 912]. No significant effects related to PD were found. See Table 1.

**TABLE 1:** Correlations between trait measures and social distancing scores.

Social Distancing	IRI/SCS	*n*	rho	*p*-value	Adjusted *p*-value (FDR = 0.05)
Behavior	EC	947	0.17	<0.001***	<0.001***
Behavior	PT	945	0.07	0.032*	0.04*
Behavior	PD	930	−0.001	0.97	0.97
Behavior	Ind	927	0.13	<0.001***	<0.001***
Behavior	Int	911	0.12	<0.001***	<0.001***
Belief	EC	947	0.22	<0.001***	<0.001***
Belief	PT	943	0.08	0.016*	0.022*
Belief	PD	930	0.05	0.1	0.14
Belief	Ind	928	0.08	0.012*	0.02*
Belief	Int	912	0.09	0.006**	0.012*

*p < 0.05; **p < 0.01; ***p < 0.001; see “Materials and Methods” section for abbreviations.

### Mask Use

Although EC and PD initially correlated significantly with Mask Use Belief, we did not find any significant correlations after correction. See Table 2.

**TABLE 2:** Correlations between trait measures and mask use scores.

Social Distancing	IRI/SCS	*n*	rho	*p*-value	Adjusted *p*-value (FDR = 0.05)
Behavior	EC	392	0.08	0.09	0.3
Behavior	PT	392	0.07	0.19	0.24
Behavior	PD	387	0.09	0.09	0.22
Behavior	Ind	387	0.07	0.16	0.23
Behavior	Int	384	0.07	0.14	0.28
Belief	EC	392	0.11	0.02*	0.1
Belief	PT	392	0.03	0.54	0.54
Belief	PD	387	0.13	0.01*	0.1
Belief	Ind	387	0.04	0.41	0.45
Belief	Int	384	0.08	0.14	0.23

*p < 0.05; see “Materials and Methods” section for abbreviations.

## Discussion

The aim of the current study was to explore if and how trait levels of empathy and self-construal style correlated with self-reported social distancing behavior and belief. We investigated this issue by collecting a large and diverse sample from five Western countries/states (i.e., Canada, United Kingdom, Sweden, New York, and Florida). Our primary analysis showed that EC and PT, both measuring different aspects of empathy, positively correlated with self-reported social distancing behavior and belief (with EC showing stronger effects). We did not find evidence to suggest that PD is related to social distancing behaviors nor belief. Our results also showed that *both* Ind and Int, measures of Independence and Interdependence Self-Construal Styles, respectively, positively correlated with self-reported social distancing behavior and belief (with stronger effects for social distancing behavior compared to belief).

Overall, these findings corroborate recent results by Pfattheicher et al. (2020) and Biddlestone et al. (2020). In regard to trait empathy, we predicted that EC would positively correlate with social distancing based on Pfattheicher et al. (2020) own empathy measure, which seemingly taps into the same aspect of empathy as EC (see section “Introduction”). This finding suggests that tapping into a person’s sense of altruism and compassion would be a very effective strategy in promoting social distancing; and indeed, Pfattheicher et al. (2020) showed that promoting empathy in participants (via having them watch a video of 91-year old man reporting that they could not visit their chronically sick wife due to the virus) increased support for social distancing relative to control conditions (also see Galea, 2020). In addition to EC, our results also showed that PT positively predicted social distancing behavior and belief. PT measures one’s ability to take another’s perspective (sometimes referred to as cognitive empathy). The strength of association between PT and social distancing behavior and belief were weaker overall weaker relative to EC; nevertheless, this result suggests that it is not only a person’s altruistic disposition that motivates them to socially distance. However, given the weak relationship between PT and social distancing, targeting altruistic tendencies seems to be the more optimal approach. Lastly, we did *not* find any association between PD and social distancing behavior and belief. Given our sample size, we are doubtful that this is due to a Type 2 error. This finding suggests that self-interest of one’s own health due to possibly feeling distress about getting the virus is not a primary motivator for socially distancing. This further suggests that tapping into people’s fears about the virus may not be very effective in motivating them to socially distance.

In regard to the trait-measures of self-construal styles, we predicted that Int would positively correlate with social distancing behavior and belief, while Ind would negatively correlate. This prediction was based on Biddlestone et al. (2020) finding that collectivism negatively correlated, and individualism positively correlated, with social distancing intentions. The positive correlation between Int and social distancing corroborates Biddlestone et al. (2020) findings, in so far as Int is considered to be analogous to collectivism at the individual level. This finding provides further support for Van Bavel et al. (2020) suggestion that we should emphasize individuals’ interdependence self-construal during times of crisis, as doing so may prioritize civic obligations and duty over personal goals and desires. Interestingly, we also found that Ind positively correlated with social distancing behavior and belief. Unlike collectivism and individualism, Int and Ind are not treated as two sides of a spectrum (Singelis, 1994; Cross et al., 2011). As such, it is not a contradiction for both self-construal styles to positively correlate with social distancing. What this suggests is that, while individualism at the cultural level predicts *less* social distancing (Biddlestone et al., 2020), it seems as though an Independent self-construal at the individual level can lead to *more* social distancing. Unlike Int, it is unclear exactly why trait-levels of Ind would predict social distancing. It is possible that, at least for some, one’s own personal aims and goals may align with social distancing; for example, if one’s work can easily be done at home, then *not* socially distancing (and possibly getting infected) would be antithetical to one’s career ambitions. However, given the surprising nature of this finding, more work will be needed to fully understand the effects of emphasizing an Independent self-construal style on social distancing.

Interestingly, we found no significant correlations between trait-levels of empathy/self-construal styles and mask neither use behavior nor belief. It is unclear why this is the case. One possibility is that due to only having data from our New York and Florida sample, we just did not have enough power to detect any real relationships. The fact that most of our results related to the social distancing measure showed quite small (but still significant) effect sizes lends credence to this interpretation. As such, future work using a larger sample size will be needed to fully explore this topic.

### Limitations

There are a number of limitations of the current study that should be noted. First, self-reported social distancing behavior and beliefs could be biased due to demand characteristics–that is, participants know they should be social distancing and, therefore, report that higher scores than what is reflected in reality. This is a general criticism of any study looking at the relationship between psychological phenomena and social distancing (e.g., Biddlestone et al., 2020; Pfattheicher et al., 2020); in lieu of objective measures of location (e.g., perhaps via GPS tracking of the participants phone–with consent of course), self-reported behavior and beliefs are the best measure available to us. It should also be noted that all the reported effect sizes, although significant, would be considered “small” based on Cohen’s guidelines; however, as Pfattheicher et al. (2020) note, in the context of a pandemic such effect sizes may still be meaningful (Funder and Ozer, 2019).

Another important limitation is that we could not meaningfully explore potential regional differences of association strength between IRI/SCS subscales and social distancing. This is primarily due to the fact that the sample size per region is small relative to the effect sizes that we obtained with our primary analysis (they are also small relative to previous work, i.e., Biddlestone et al., 2020; Pfattheicher et al., 2020). This means that any inconsistencies of results between regions could simply be due to a lack of power to detect an effect; and indeed, for our smallest effects (e.g., rho = 0.09), G^∗^power suggests that a sample size of >900 is needed to obtain 80% power to detect them. As such, even if we were to find and report significant differences between regions, it would be unclear whether such differences were due to country-specific cultural differences or simply due to sampling error as a result of a lack of power. As such, future cross-national research should consider using large enough sample sizes to detect these effects in order to appropriately compare them.

Finally, it should be noted that there are no doubt numerous factors that may influence an individual’s decision to socially distance/wear masks that were not measured in the current study (e.g., political orientation, SES, beliefs in conspiracy theories, education, etc.). Indeed, although we collected a rather large and diverse sample (at least relative to a commonly used student sample), we are still limited by the fact that our sample consists of participants from developed nations in the western world. Of course, any single study will always be limited by the number of available resources; ultimately, the current study simply contributes a small piece to our overall understanding of the psychological factors that influence social distancing specifically, and human behavior during a global health crisis more generally. Future theoretical and meta-analytic work synthesizing the growing number of papers exploring this topic will ultimately be needed to fully explicate our understanding of these issues.

## Conclusion

In conclusion, the current study showed that PT and EC, two different dimensions of trait-empathy, positively correlates with self-reported levels of social distancing behavior and belief; however, PD, a measure of self-distress, did not. These findings suggest that promoting and emphasizing empathy, as opposed to self-distress (via the harmful effects of the virus to one’s own health), may be an effective strategy is increasing social distancing from the population. We also found that both independence and interdependence self-construal styles positively correlates with social distancing behavior and belief. While the former result is in line with recent suggestions of emphasizing group unity and civic obligations to increase social distancing in the population, the latter result is both surprising and counter-intuitive. We suggested that more work is ultimately needed to fully explain why and how an emphasis on Independent self-construal can lead to more social distancing. Lastly, we noted a number of limitations of this study that makes strong interpretations of the results difficult, as well as suggested future avenues of research to better explore these topics.

## Data Availability Statement

The raw data supporting the conclusions of this article will be made available by the authors, without undue reservation.

## Ethics Statement

The studies involving human participants were reviewed and approved by McMaster Research Ethics Board. The patients/participants provided their online informed consent to participate in this study.

## Author Contributions

CG: conceptualization, methodology, software, formal analysis, investigation, writing–original draft, writing–review and editing, and visualization; DJ: conceptualization, methodology, formal analysis, writing–review and editing, and visualization; SO: conceptualization, methodology, resources, writing–review and editing, supervision, and funding acquisition. All authors contributed to the article and approved the submitted version.

## Conflict of Interest

The authors declare that the research was conducted in the absence of any commercial or financial relationships that could be construed as a potential conflict of interest.

## Footnotes

1 https://www.who.int/news-room/detail/29-06-2020-covidtimeline

2 For example: https://www.bbc.com/news/world-us-canada-52417610
